# The consequences of a year of the COVID-19 pandemic for the mental health of young adult twins in England and Wales

**DOI:** 10.1192/bjo.2022.506

**Published:** 2022-07-21

**Authors:** Kaili Rimfeld, Margherita Malanchini, Ryan Arathimos, Agnieszka Gidziela, Oliver Pain, Andrew McMillan, Rachel Ogden, Louise Webster, Amy E. Packer, Nicholas G. Shakeshaft, Kerry L. Schofield, Jean-Baptiste Pingault, Andrea G. Allegrini, Argyris Stringaris, Sophie von Stumm, Cathryn M. Lewis, Robert Plomin

**Affiliations:** Social, Genetic and Developmental Psychiatry Centre, Institute of Psychiatry, Psychology and Neuroscience, King's College London, UK and Department of Psychology, Royal Holloway University of London, London, UK; Social, Genetic and Developmental Psychiatry Centre, Institute of Psychiatry, Psychology and Neuroscience, King's College London, and Department of Psychology, Queen Mary University of London, UK; Social, Genetic and Developmental Psychiatry Centre, Institute of Psychiatry, Psychology and Neuroscience, King's College London, and National Institute for Health Research (NIHR) Maudsley Biomedical Research Centre, South London and Maudsley NHS Trust, London, UK; Social, Genetic and Developmental Psychiatry Centre, Institute of Psychiatry, Psychology and Neuroscience, King's College London, UK; Clinical, Educational & Health Psychology, Division of Psychology & Language Sciences, Faculty of Brain Sciences, University College London, UK; Mood, Brain & Development Unit, Emotion and Development Branch, National Institute of Mental Health, Bethesda, MD, USA; Psychology in Education Research Centre, Department of Education, University of York, UK; Social, Genetic and Developmental Psychiatry Centre, Institute of Psychiatry, Psychology and Neuroscience, King's College London, NIHR Maudsley Biomedical Research Centre, South London and Maudsley NHS Trust, London, and Department of Medical and Molecular Genetics, King's College London, UK; Social, Genetic and Developmental Psychiatry Centre, Institute of Psychiatry, Psychology and Neuroscience, King's College London, London, UK

**Keywords:** Mental health, COVID-19, lockdown, young adults, pandemic

## Abstract

**Background:**

The COVID-19 pandemic has affected all our lives, not only through the infection itself but also through the measures taken to control the spread of the virus (e.g. lockdown).

**Aims:**

Here, we investigated how the COVID-19 pandemic and unprecedented lockdown affected the mental health of young adults in England and Wales.

**Method:**

We compared the mental health symptoms of up to 4773 twins in their mid-20s in 2018 prior to the COVID-19 pandemic (T1) and during four-wave longitudinal data collection during the pandemic in April, July and October 2020, and in March 2021 (T2–T5) using phenotypic and genetic longitudinal designs.

**Results:**

The average changes in mental health were small to medium and mainly occurred from T1 to T2 (average Cohen d = 0.14). Despite the expectation of catastrophic effects of the pandemic on mental health, we did not observe trends in worsening mental health during the pandemic (T3–T5). Young people with pre-existing mental health problems were disproportionately affected at the beginning of the pandemic, but their increased problems largely subsided as the pandemic persisted. Twin analyses indicated that the aetiology of individual differences in mental health symptoms did not change during the lockdown (average heritability 33%); the average genetic correlation between T1 and T2–T5 was 0.95, indicating that genetic effects before the pandemic were substantially correlated with genetic effects up to a year later.

**Conclusions:**

We conclude that on average the mental health of young adults in England and Wales has been remarkably resilient to the effects of the pandemic and associated lockdown.

The COVID-19 pandemic has affected both those infected by the virus and those spared from the infection who had to adhere to strict lockdowns. The COVID-19 pandemic has changed the everyday lives of all, through full or partial forced isolation (lockdown), closure of schools and public spaces, and associated economic consequences. Several studies have reported that the pandemic negatively affected mental health, which seems reasonable given the scale of the crisis.^1–4^ This pandemic might worsen mental health on average in the population, but young adults are likely to be affected disproportionately more.^5–8^ Indeed, young adults' mental health has been reported to be worse following the pandemic-related lockdown compared with the rest of the adult population even after the first restrictions were lifted.^9^

Young adulthood (the period between the early 20s and early 30s) is a time marked by instability and fragility in several aspects of life and is a critical age for the development of psychopathologies.^10–12^ Even before COVID-19, the Labour Force Survey of the Office for National Statistics reported that 17.9 M days of work were lost every year to stress, depression and anxiety.^13^ The ~12 M young adults living in the UK make up over a third of the current workforce and will account for nearly half of the workforce within the next two decades. The instability and financial hardship generated by the COVID-19 pandemic are likely to exacerbate the struggles of young adults, with cascading effects on the health, well-being and economic stability of the nation.

Although several studies have reported that the pandemic negatively affected mental health, the extent of these effects remains an open question, because most reports have focused on statistically significant average differences rather than effect size. When reported, the effect sizes are typically small to medium,^14^ and some studies have found little change in mental health following the COVID-19 pandemic and lockdown.^15–18^ Some reports suggest that population levels of anxiety and depression were elevated immediately after lockdown but then decreased again as the pandemic continued.^14,16^ There is also some evidence for improvement in mental health symptoms during the pandemic.^19,20^ For example, some people seemed to thrive during the pandemic and lockdown, reporting increased well-being.^21,22^ These seemingly inconsistent findings might be due to methodological differences between studies, such as differences in age, measures, timing of the lockdown and whether pre-pandemic measures were available.

Notably, the majority of the research to date has focused on average psychological changes during the COVID-19 crisis and the experience of lockdown, but this crisis is likely to have affected individuals differently.^23^ Individual differences are likely to include both negative changes such as increased anxiety and depression and positive changes such as increased well-being,^22^ which could cancel out average changes. For this reason, it is important to study individual differences as well as average differences (means) in response to the pandemic and the experience of lockdown.

A fundamental aspect of individual differences that is often ignored when studying the response to the COVID-19 crisis is genetic variation. Yet, it has been demonstrated over decades of twin studies that inherited DNA differences contribute substantially to most psychological traits.^24,25^ Moreover, research has shown that sensitivity to environmental changes has a heritable component.^26^ That is, individuals differ considerably in how they respond to similar environmental situations, both adverse environmental conditions, such as the pandemic, or protective, favourable environmental conditions, such as a nurturing home environment. These individual differences in the responses to environmental conditions have been shown to be highly heritable.^26^ It is therefore reasonable to assume that individual differences in the response to the COVID-19 crisis are also partially driven by genetic variation between individuals.

Our first study,^27^ using the Twins Early Development Study (TEDS) sample, compared a wide range of psychological measures (30 traits) from T1 (2018) to T2 (April 2020) and found modest and unsystematic mean changes in these traits after 1 month of the COVID-19 lockdown. The largest negative effects of the pandemic were reduced volunteering (d = 0.84), decreased achievement motivation (d = 0.47) and increased hyperactivity-inattention (d = 0.42). However, we also observed many positive changes in response to the pandemic, most notably reduced peer victimisation, reduced alcohol (quantity) consumption and decreased self-harming. It is possible that 1 month of lockdown was an insufficient time frame to examine the negative consequences of these unprecedented restrictions. Furthermore, the lockdowns did not end after 1 month, and restrictions continued until March 2021; it is therefore important to study the longer-term effects of the COVID-19 pandemic on mental health.

Our first study also examined the aetiology of mental health symptoms before and 1 month after the lockdown. We found that genetic factors accounted for around half of the reliable variance in diverse psychological traits both at T1 and T2. Furthermore, we found that genetic factors at T1 were highly correlated with genetic factors at T2. We also investigated possible moderators, for example, family socioeconomic factors, living conditions and financial difficulties, but found no evidence of gene–environment interaction. Here, we investigate the extent to which the aetiology of mental health symptoms changes over a longer period, from T1 to T5.

We used longitudinal data collected over five waves (2018 and four pandemic waves of assessment 1, 4, 7 and 11 months after the first lockdown in Britain began in March 2020). The sample consisted of twins in TEDS^17^ assessed when the twins were in their mid-20s. Our focus was on tracking individual differences in mental health trajectories over the course of the crisis compared with 2018 using diverse measures of mental health symptoms, including conduct problems, emotional problems, hyperactivity, peer problems, prosocial behaviour, general anxiety, depression and self-harm. The genetic and environmental origins of these measures were assessed by the classical twin method that compares the resemblance of identical and non-identical twins. It is reasonable to expect that a major environmental shift, such as the COVID-19 pandemic, would reshuffle individual differences. For example, the genetic effects could be either reduced or amplified; that is, the heritability of mental health symptoms could increase or decrease but still be explained by the same genetic factors. Alternatively, this environmental shift could evoke new/innovative genetic effects.^28–30^ In addition, we investigated factors influencing changes in mental health using genome-wide polygenic scores (GPS), aggregated scores that capture genetic predisposition towards risk and protective factors based on previous genome-wide association studies (GWAS),^31^ which allowed us to establish whether individuals at higher risk of psychiatric diseases were disproportionately affected by the COVID-19 pandemic. We also investigated the extent to which family socioeconomic status, lockdown conditions, life changes and home environment affected response to the pandemic.

Several studies have indicated that lockdown measures could be especially detrimental for young adults with existing psychological and psychiatric vulnerabilities.^8,22,32^ Here, we capitalised on data collected in TEDS in T1 (2018) to identify young adults who had experienced mental health problems prior to the COVID-19 pandemic. We assessed the extent to which these individuals were disproportionately affected by the pandemic and associated lockdown. We also addressed this issue using polygenic scores as indicators of genetic vulnerabilities.

Our hypotheses and plan for analyses were preregistered in the Open Science Framework (OSF; https://osf.io/gzrk3) prior to accessing the data. Our main hypotheses were that mean changes from T1 to T5 would be significant, with small-to-modest effect sizes for the entire sample but larger in vulnerable groups. Heritability was predicted to be substantial at each assessment from T1 to T5. We also hypothesised that mental health changes from T1 to T5 would show significant but modest genetic influence, with most of the changes environmental in origin. Polygenic scores were predicted to yield a similar pattern of results as the twin analyses.

## Method

### Sample

Our sample consisted of young adults enrolled in TEDS,^33^ a twin study that recruited twins born between 1994 and 1996 in England and Wales, as identified through birth records. Invitations were sent to families by the UK Office for National Statistics after screening for infant mortality, and 16 810 families expressed interest in taking part. TEDS conducted the first wave of data collection when twins were around 18 months old, including demographic information, data about pregnancy and childbirth, and questions related to zygosity. The sample was, and remains, reasonably representative of the population in England and Wales in terms of ethnicity and socioeconomic factors for this birth cohort.^33^

The current study used data from the TEDS data collection wave conducted when the youngest twins were aged 21 (completed in 2018), together with COVID-19 data collection completed in April, July and October 2020, and in March 2021. The sample includes up to 4773 unrelated individuals (one randomly selected twin per pair), up to 1501 complete monozygotic twin pairs and up to 2380 complete dizygotic twin pairs; these sample sizes vary across measures and measurement occasions (Supplementary Table 1 available at https://doi.org/10.1192/bjo.2022.506). All available data were used.

### Ethical approval

Ethical approval was provided by the King's College London Research Ethics Committee (reference number: PNM/09/10-104), and informed written consent was received from all participants.

### Measures

**Fig. 1:**
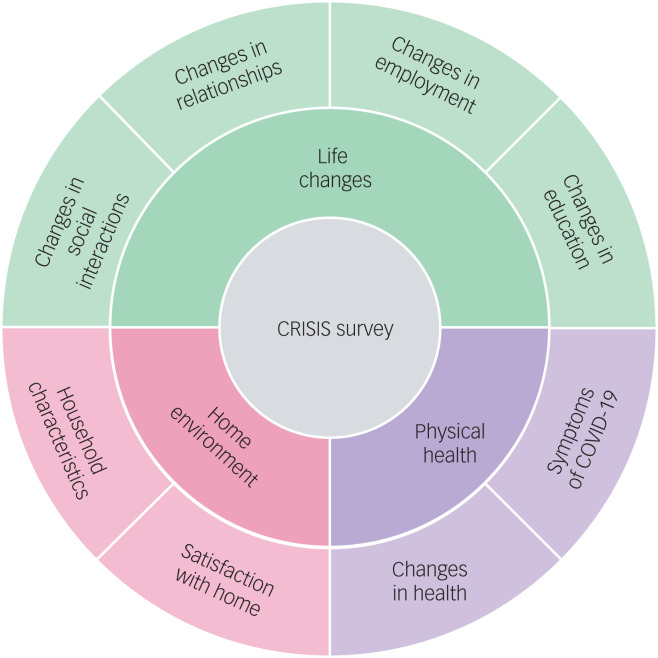
Summary of the constructs measured by the CRISIS questionnaire.

In 2018, when the twins were aged 21–25 years (mean age = 22.27 years, s.d. = 0.9), they completed a battery of mental health measures, which are listed in Table 1. These served as a baseline to examine how the COVID-19 crisis has changed the mental health of young adults during the four subsequent waves of assessment. The twins completed the same battery of mental health measures in April 2020 (1 month after the first COVID-19 lockdown began), July 2020, October 2020, and March 2021. In addition, the CoRonavIruS Health Impact Survey (CRISIS)^34^ was administered at T2–T5. CRISIS assesses mental health, behaviours, home environment, physical health and life changes (Fig. 1).

**Table 1:** Measured variables

Theme	Variable name	Scale/item^a^	No. of items	Reference^b^
CRISIS questionnaire	Multiple items	Items about home environment, life changes, and physical health	41	Adapted from CRISIS; Nikolaidis et al (2020)^34^
Mental health	Conduct problems	SDQ – conduct problems^c^	5	Goodman (1997)^68^
	Emotional problems	SDQ – emotional problems	5	Goodman (1997)^68^
	Hyperactivity	SDQ – hyperactivity	5	Goodman (1997)^68^
	Peer problems	SDQ – peer problems	5	Goodman (1997)^68^
	Prosocial behaviour	SDQ – prosocial behaviour	5	Goodman (1997)^68^
	General anxiety	General anxiety: the Severity Measure for Generalised Anxiety Disorder	10	Craske et al (2013)^69^
	Depression	Short Mood and Feelings Questionnaire	8	Angold et al (1995)^70^
	Behavioural Problems	SDQ – combined score of conduct problems, emotional problems, hyperactivity and peer problems subscales		Goodman (1997)^68^
	Self-harm	CASE – self-harm	1	Adapted from Madge et al (2008)^71^

SDQ, Strength and Difficulties Questionnaire; CASE, the Child & Adolescent Self-harm in Europe questionnaire.

a. More information about the measures and the references can be found in the TEDS data dictionary.

b. Note that measures were shortened and adapted from the referenced measures.

c. The SDQ was shortened from 13 to 8 items, with the addition of 1 quality control item, by TEDS researchers prior to data collection.

All data were self-reported and were collected using online questionnaires, or there was also an option to contribute to the data collection via the paper and pencil questionnaires (for data collection in 2018 only). More information is available at the TEDS Data Dictionary (https://www.teds.ac.uk/datadictionary/home.htm).

### Genotyping

DNA samples were obtained from 12 500 individuals in the TEDS sample and genotyped on one of two DNA microarrays (Affymetrix GeneChip 6.0 or Illumina HumanOmniExpressExome chips). After stringent quality control, the total sample size available for genomic analyses was 10 346 (including 7026 unrelated individuals and 3320 additional dizygotic co-twins). Of these, 7289 individuals were genotyped using Illumina arrays, and 3057 individuals were genotyped using Affymetrix arrays^33^ (see Ref. 35 for a detailed description of genotyping and quality control).

### Polygenic scores

DNA was used to calculate polygenic scores to serve as an index for genetic vulnerabilities for psychiatric diseases. We constructed GPS using LDpred,^36^ which corrects GWAS summary statistics for local linkage disequilibrium (LD). See Allegrini et al^37^ for a detailed description of LDpred analytic strategies used in calculating GPS in the TEDS sample. Here, we used fraction 1.0 for all GPS. This fraction of causal markers has been shown to be the most predictive for psychiatric traits.

The following GWAS summary statistics were used to construct the GPS: schizophrenia,^38^ bipolar disorder,^39^ depression,^40^ anxiety,^41^ attention-deficit hyperactivity disorder,^42^ autism spectrum disorder,^43^ EA3 (educational attainment),^44^ risk-taking sensitivity,^45^ cross-disorder (combining eight psychiatric disorders),^46^ obsessive–compulsive disorder,^47^ post-traumatic stress disorder,^48^ neuroticism^49^ and Insomnia.^50^ These summary statistics were selected because they represent the most powerful GWAS for behavioural traits at the time of the analyses. Note that some summary statistics in the original GWAS contained 23andMe data. The summary statistics employed in the present study did not include 23andMe data; these data had to be removed due to 23andMe's data availability policy.

In addition to examining the genetic vulnerabilities of each GPS, we also used the first principal component of the 11 psychiatric GPS (excluding EA3 to limit the final score to psychiatric traits, as well as cross-disorder GWAS summary statistics as these already include psychiatric disorders included in this composite measure) to index general genetic vulnerability to behavioural problems. This approach comes with limitations, mainly the sample overlap between some of the GWAS (mostly from shared controls), which may inflate the correlation between the GWAS results. It has been shown, however, that different methods used to derive a genomic *p* factor yield similar results.^51^

### Statistical analyses

We compared the self-reported mental health symptoms of up to 4773 twins in their mid-20s in 2018 prior to the COVID-19 pandemic (T1) and during a four-wave longitudinal data collection process during the pandemic in April, July and October 2020 and in March 2021 (T2–T5), using phenotypic and genetic longitudinal designs.

Our statistical analysis plan was registered with the OSF prior to accessing the data. Scripts have been made available on the OSF site. All analyses were completed using SPSS Statistics software^52^ and R version 4.0.^53^

#### Phenotypic analyses

We compared the average differences in these traits from T1 to T2–T5 by comparison of means and s.d. at T1–T5; this was also done separately for males and females. Multivariate analysis of variance (MANOVA) was used to test the significance of mean differences between time points and differences between males and females at all time points, as well as to test interactions between time and sex.

As significant though small sex differences emerged, we corrected all scores for mean sex differences using the regression method. Correcting for sex and age is important in the analysis of twin data because members of a twin pair are identical in age and identical twins are identical for sex, which, if uncorrected, would inflate twin estimates of shared environment.^54^ These age- and sex-adjusted standardised residuals were used in all subsequent analyses.

Several variables were skewed. In our previous study,^55^ we transformed the data to attenuate skew but found that the results were highly similar to those using untransformed data. For this reason, we used untransformed data in our analyses here.

We used latent growth curve (LGC) models to extract stable individual differences from before the COVID-19 pandemic started (T1), evaluate individual differences at the starting point (2018 data collection) and extract the rate of change over the pandemic (T2–T5) for the same traits. We fitted LGC models for all mental health measures separately, testing for linear, quadratic and piecewise trends in the data. We used the R package lavaan for LGC (on R version 1.4 for Mac OS). FIML (full information maximum likelihood) was used to account for missing data.^56^

In addition, we conducted latent profile analyses (LPA) to identify any sub-populations in our sample that responded differentially to the COVID-19 pandemic. LPA was fitted to each of the mental health measures separately. LPA is a statistical method for identifying homogenous subgroups of individuals based on a set of continuous measured variables called indicators. Classification of individuals into latent classes is probabilistic, and LPA allows for selection of the optimum numbers of classes (the optimum model) through comparison of model fit indices. Longitudinal LPA is a special case of LPA where the indicators consist of repeated measures of the same variable across time (T1–T5). For each outcome (symptoms of mental health; Table 1), models with stepwise increases in numbers of classes, from two to seven classes, were fitted. In all models, variances were equated and covariances fixed to 0, and missing values across timepoints were imputed (single imputation using the mix package alongside the tidyLPA package on R version 1.4 for Mac OS). We used the tidyLPA package,^57^ which draws on the functionality of mclust,^58^ in R 3.6.2 for all analyses.

#### Genetic analyses

The classical twin design was used to assess genetic and environmental contributions to individuals’ traits at each wave of assessment. The twin method capitalises on the genetic differences between twins – that is, it compares monozygotic (identical) twins, who are 100% similar genetically, with dizygotic (non-identical) twins, who share on average 50% of their segregating genes. Environmental factors that make members of twin pairs similar to each other are defined as shared environmental factors, and environmental factors that do not contribute to similarities between twin pairs are defined as non-shared environmental factors. Using these family relatedness coefficients, it is possible to estimate the relative influence of additive genetic, shared environmental and non-shared environmental effects on the variance and covariance of phenotypes by comparing intraclass correlations for monozygotic and dizygotic twins.^24^ In the model, non-shared environmental variance also included any measurement error. These parameters can be estimated more accurately using structural equation modelling (SEM), which also provides 95% confidence intervals and estimates of model fit. The SEM program OpenMx for Mac OS was used for all twin model-fitting analyses.^59^

The univariate model can be extended to a multivariate model to investigate the aetiology of the covariance between two traits. This method also enables the estimation of the genetic correlation, indicating the extent to which the same genetic variants influence two phenotypes. The shared environmental correlation and non-shared environmental correlation can also be estimated.^24,60^ A correlated factor solution was used to calculate genetic correlations between mental health measures from T1–T5. Cholesky decomposition analysis, which is conceptually similar to hierarchical regression, was used to estimate the extent to which genetic and environmental effects at T2, T3, T4 and T5 were independent of T1, indicating the aetiology of changes in mental health symptoms during COVID-19. We conducted these analyses for each mental health measure.

GPS were used to estimate the variance explained in mental health measures from T1 to T5 using linear regression adjusted for sex, age, the first ten PCs, genotyping batch effects and genotyping chip effects. In our preregistered plan, we had specified that we would use GPS to predict both intercept and slope in mental health traits (LGC model), but as we did not find significant slope in the models, we were not able to complete this step in our analyses.

#### Analysis of extremes

To assess whether extreme groups were differentially affected by the COVID-19 pandemic and associated lockdown, we compared extreme groups, defined as less than and greater than 1 s.d. of the standardised scores for the following variables. (a) Socioeconomic status (SES) as assessed when twins joined TEDS in infancy; this was stable across development, with a correlation of 0.71 between SES at first contact and SES collected when twins were 16 years old. (b) The phenotypic psychopathology factor, that is, the first principal component of mental health for data collected (Table 1) in 2018 using a principal component analysis (PCA) approach; see Allegrini et al^61^ for details on the PCA approach for mental health measures using the same data. (c) Genomic psychopathology factors, that is, the first principal components of all psychiatric polygenic scores); depression,^40^ anxiety,^41^ risk-taking sensitivity,^45^ cross-disorder^46^ and educational attainment^44^ were analysed separately, as these GPS were closely related to the phenotypes studied here.

#### Environmental correlates of COVID-19 lockdown

We also studied the association between mental health traits from T2–T5 and environmental factors (e.g. lockdown conditions), physical health (e.g. COVID-19 symptoms, short- and long-term) and life changes (e.g. mental health or financial worries) using CRISIS measures (Fig. 1). In our preregistered analyses plan, we intended to test whether CRISIS measures predicted intercept and slope in mental health; however, we did not detect significant linear slope for any mental health measures. Therefore, we examined the effects of environmental extremes on mental health outcomes during the COVID-19 pandemic and lockdown. For example, we compared low versus high (±1 s.d.) family SES obtained when the twins were infants. For quantitative measures (e.g. worries about mental health or financial well-being) we reported the phenotypic correlations.

## Results

### Descriptive statistics for mental health from T1 to T5

**Fig. 2:**
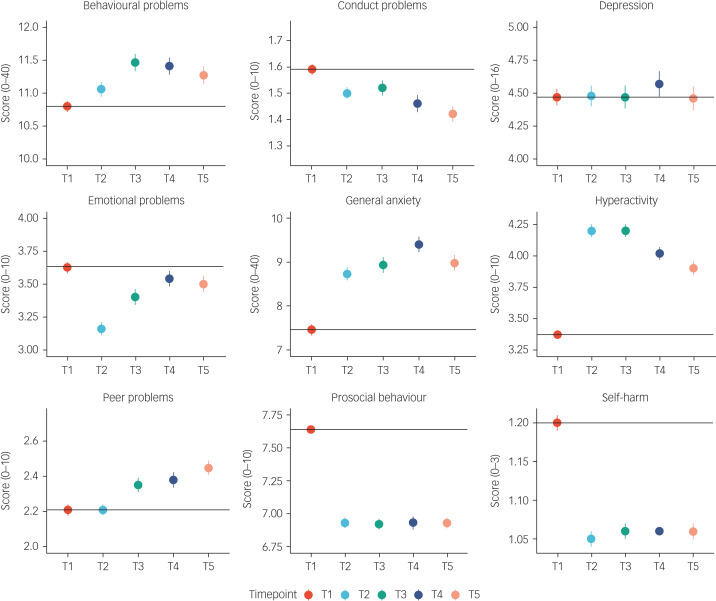
Descriptive statistics (mean and s.e.) for all mental health measures from T1 to T5.

Figure 2 illustrates the means and s.e. for the mental health measures from T1 to T5. The average changes in mental health symptoms were small to medium (the average partial eta squared from T1 to T5 was 0.026) and mainly occurred over the 2 year period from 2018 to March 2020 (average Cohen's d = 0.14 from T1 to T2), with no evidence of average worsening mental health as the pandemic persisted. The largest negative effects on mental health were increased (worsening) general anxiety (Cohen's d = 0.17 from T1 to T2), increased hyperactivity (Cohen's d = 0.37) and decreased prosocial behaviour (Cohen's d = 0.36). These worsening mental health effects were considered to be small to medium effect sizes (a Cohen's d of 0.35 accounted for 3% of the variance).^62^ For anxiety and prosocial behaviour, the negative effect (worse symptoms on average) persisted from T2 to T5; for hyperactivity, the effect decreased with time. Moreover, some changes were positive for mental health: conduct problems, emotional problems and self-harm decreased. However, these small to medium effect sizes based on Cohen's criteria were still meaningful.^63^ Based on current evidence, an effect size of 0.3 for psychiatric traits is considered to be medium to large, similar to that expected after natural disasters (e.g. earthquake).^64^

These descriptive statistics were based on one randomly selected twin per pair, but the results were virtually identical when the analysis was repeated with co-twins. Details of descriptive statistics results from MANOVA are included in Supplementary Table 1. On average, females reported more mental health problems than males, but these mean sex differences only explained on average 3% of the variance in these measures (average partial eta squared of 0.035). There were no significant time–sex interactions. Descriptive statistics for males and females separately, and MANOVA results, are presented in Supplementary Table 1.

Supplementary Table 2 presents descriptive statistics for CRISIS measures from T2 to T5. On average, rates of possible COVID-19 symptoms were slightly lower at the start of the pandemic compared with later data collection points; however, worries (about COVID-19, mental health and finances) were slightly elevated at the start of the pandemic and declined slightly as the pandemic continued.

In our preregistered analysis plan (https://osf.io/gzrk3), we specified LGC models to estimate individual differences at the starting point (2018 data collection, T1) and individual differences in the rate of change that occurred over time for the same traits (slope from T1 to T5). We predicted that there would be a significant linear slope from T1 to T5 for all mental health measures. We had plans to test whether GPS and CRISIS measures (COVID worries, mood states, life changes during the pandemic) predicted intercept and slope for mental health symptoms. However, although the LGC showed a significant change (slope) from T1 to T5 (see Supplementary Table 3 for results), the variance in this slope was very small. In addition, the linear trend across T1–T5 did not really fit the data well, given the rupture that occurred at T2 for many phenotypes. It was therefore not reasonable to predict the change from T1 to T5. The quadratic trends in the data fitted better than the model with just one slope. We could not test piecewise trends in the data, as there was only one data point before change happened in T2, and a minimum of three data points is required to appropriately estimate a linear slope. We decided not to predict the change from T1 to T2 for several reasons. (a) It was uncertain whether changes over a 2 year period were due to the pandemic, as the maturation hypothesis could fit equally well. (b) The increase only occurred between T1 and T2; we did not observe worsening of mental health symptoms over the pandemic. (c) The predictor variables (COVID-19 worries, mood states, life changes during the pandemic) were collected from T2; therefore, they were not applicable for prediction of change from T1 to T2. We opted to use latent profile analyses (LPA) instead to check for the presence of subgroups in each mental health outcome.

### Latent profile analyses

**Fig. 3:**
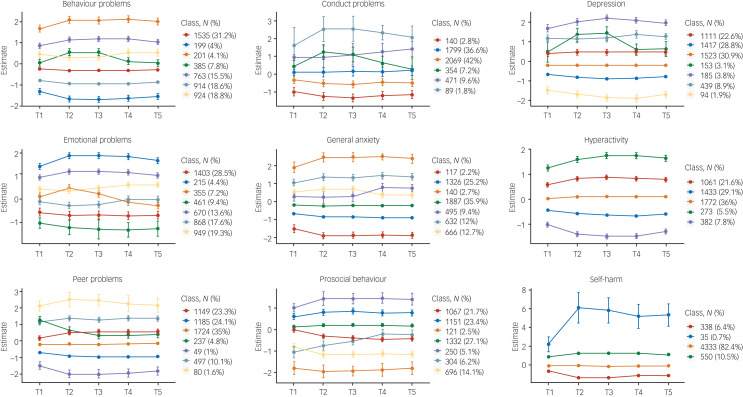
Latent profile analyses presenting the optimum model for each mental health outcomes (95% CI as error bars).

LPA, using standardised measures of mental health symptoms at each time point (mean of 0 and s.d. of 1), was in agreement with the descriptive statistics showing little change in mental health symptoms during the COVID-19 pandemic. LPA identified subgroups based on the quantitative trait measures in our study. For most measures, the optimal model identified seven profiles. Most differences across profiles were due to changes from T1 to T2; the profiles did not change much from T2–T5. Most of the sample were in the middle profiles, as illustrated in Fig. 3, which showed no changes in mental health symptoms. The sample sizes for the profiles showing an increase or decrease in mental health were small, as was the change in mental health symptoms (less than half a s.d. on average). For example, for hyperactivity, individuals in latent profile 2 (5.5% of the sample) showed an increase in symptoms from T1 to T2, and this remained elevated through T3, T4 and T5. Similarly, profile 3 (21.6% of the sample) showed an increase from T1 to T2, but the symptoms returned to pre-pandemic level. Model fit statistics for all LPA are presented in Supplementary Table 4.

Although self-harm dramatically increased for one latent class, there were only 35 individuals (fewer than 1% of the sample) in this profile. In addition, this measure was highly skewed. For these reasons, we did not perform additional analyses with the self-harm measure, although we plan to study the predictors of self-harm and its changes in our future research.

### Individual differences

Small unsystematic mean differences, as well as latent profiles, could mask large individual differences. Supplementary Fig. 1 presents the pattern of individual variability in mental health measures from T1 to T5. There were large individual differences at every data collection point, as well as individual differences in changes in mental health measures during the COVID-19 pandemic. The overall (mean) trajectories are presented in black in the figure (similar to the means in Fig. 2) and showed no change from T1 to T5, except for hyperactivity and prosocial behaviour, which change for the worse from T1 to T2.

If there were large differences in how individuals responded to the COVID-19 pandemic and associated lockdown then we might expect increased variance. We have already reported in a previous study that we did not observe variance differences from T1 to T2.^27^ Here, we found that the variance remained similar from T1 to T5 (see Supplementary Table 1 for standard deviations across measures from T1–T5). We did not observe an increase in variance following the COVID-19 crisis.

**Fig. 4:**
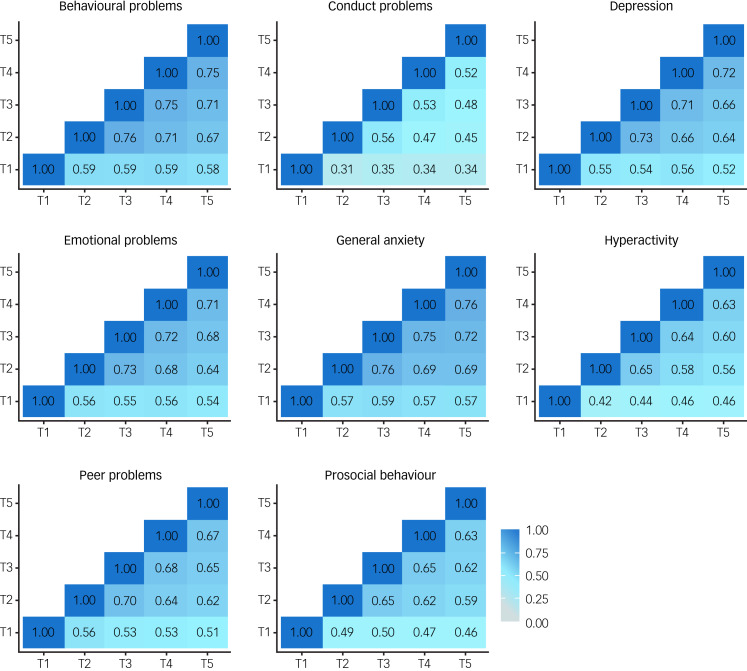
Phenotypic correlations across time points.

In addition, if COVID-19 re-shuffled individual differences in mental health symptoms then we would expect to see little correlation between mental health symptoms from T1 to T5. However, in our previous report, we noted substantial correlations between measures across the 3 years from T1 to T2 (average correlation = 0.59). Here, we found even larger correlations from T2 to T5 (Fig. 4), which had an average interval of 3 months (average correlation = 0.65). The 3 month interval between T2 and T3 was (average correlation of 0.69) comparable with the average 2 week test–retest reliability from the TEDS preparatory work for our 2018 (T1) assessment of the twins (average test–retest reliability = 0.75).

See Supplementary Table 5 for correlation coefficients with 95% confidence intervals.

### Genetic analyses

**Fig. 5:**
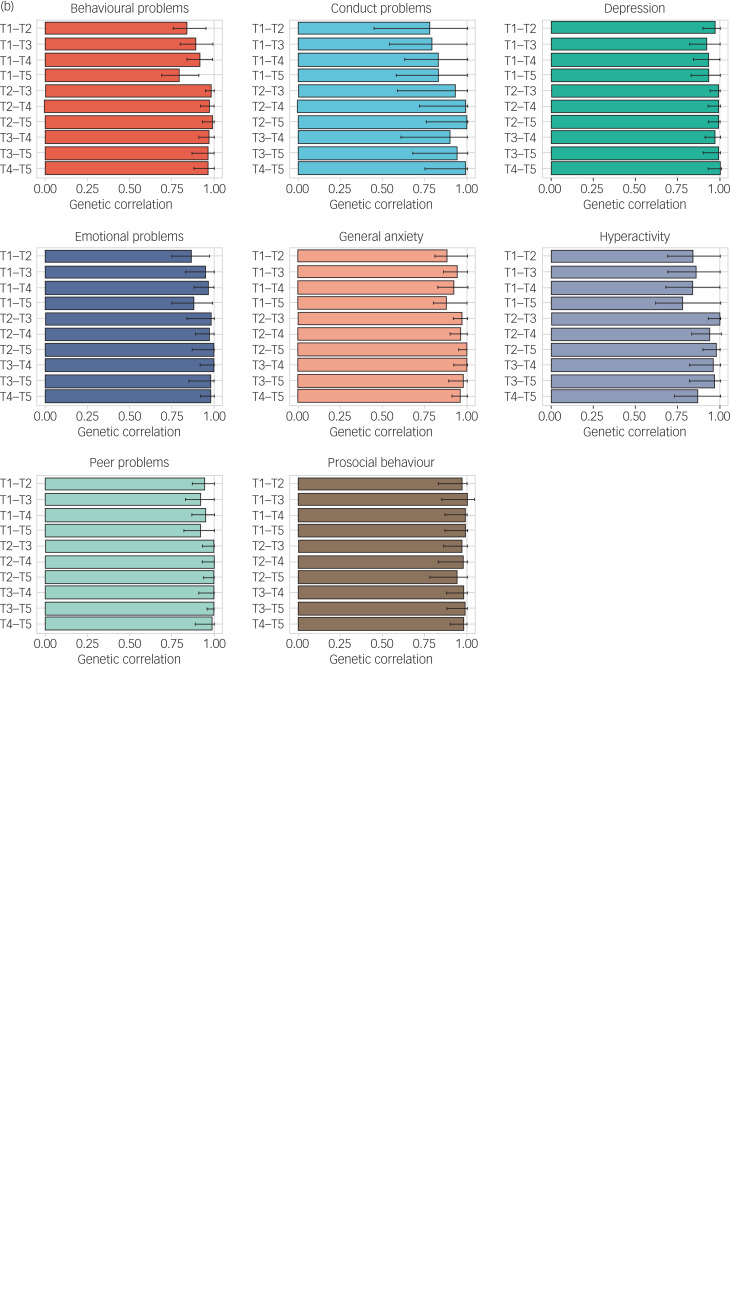
(a) Univariate twin model-fitting results showing the heritability of traits across time points (with 95% confidence intervals). (b) Genetic correlations across time points with 95% confidence intervals.

As illustrated in Fig. 5(a), twin estimates of heritability for mental health measures were on average 33%, with the majority of the variance explained by non-shared environmental components. Importantly, the heritability of mental health symptoms studied here did not change systematically despite the COVID-19 pandemic and lockdown. One exception was conduct problems, which only showed significant heritability at T1 and T4, although the heritability estimates from T1 to T5 had overlapping confidence intervals. Full univariate model-fitting results with 95% confidence intervals are presented in Supplementary Table 6. Twin model-fitting also showed that variance did not increase from T1 to T5, as indicated by the unstandardised variance components (Supplementary Fig. 2).

The most striking genetic result was that the average genetic correlation was 0.95 between T1 and T2–T5 (Fig. 5(b)). The genetic correlation indexes the correlation of genetic effects between two time points, which is independent of the heritability of the traits. For example, although the heritability of conduct problems was not significantly different from zero at T2, the genetic correlation between T1 and T2 for conduct problems was not significantly different from unity. Non-shared environmental correlations were moderate, with an average correlation of 0.41, indicating that the non-shared environmental factors that explained individual differences in mental health symptoms at one time were correlated with environmental factors at another time during the COVID-19 pandemic. (See Supplementary Table 7 for genetic, shared and non-shared environmental correlations with 95% confidence intervals.) Supplementary Table 8 presents the results of the multivariate Cholesky analyses, which showed that there was negligible genetic variance at T2–T5 independent of genetic variance at T1.

### Polygenic score analysis

We also calculated the variance explained by all polygenic scores (see Methods). We hypothesised that GPS would significantly predict variance in mental health measures; however, although some of the models remained statistically significant after multiple testing corrections, many were not, and the average prediction was low. On average, the polygenic scores (both genomic *p* factor and individual GPS) explained less than 1% of the variance in mental health measures; importantly, the variance explained did not change from T1 to T5. See Supplementary Tables 9–14 for the results of GPS prediction longitudinally from T1 to T5.

### Extremes analyses of individuals with pre-existing mental health problems

**Fig. 6:**
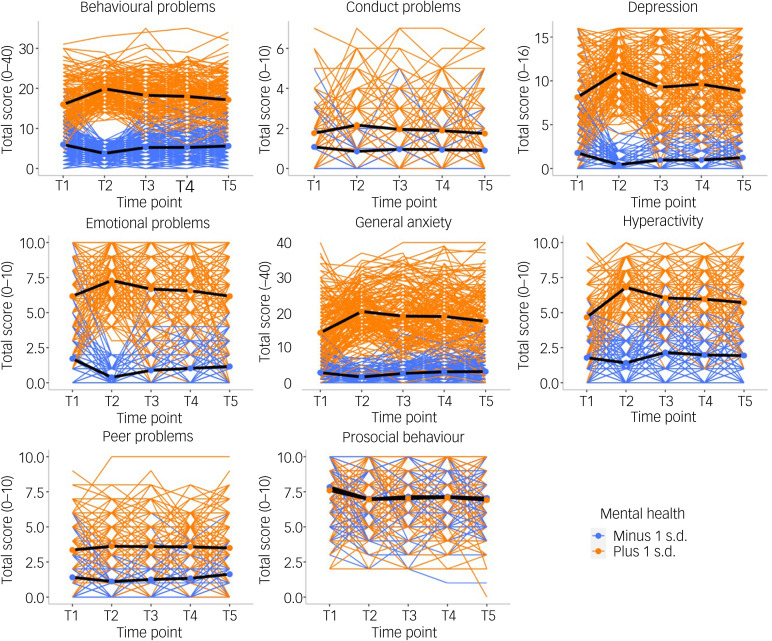
Patterns of individual variability across T1–T5 for all mental health measures separated by ±1 s.d. on *p* factor at T1 prior to the start of the pandemic. Individual trajectories are presented as coloured lines, and the average mean trajectory is shown as a black line.

We compared two groups of individuals: those with pre-existing mental health problems (+1 s.d. on the first principal component of mental health problems in 2018; *N* = 265−429; see Supplementary Table 15 for details) and those who had lower than average levels of mental health problems before the pandemic started (−1 s.d. on the first principal component of mental health problems in 2018; *N* = 227−329; see Supplementary Table 15 for details). As illustrated in Fig. 6, individuals with pre-existing mental health problems reported increased problems at T2 for most measures (average d = 0.47 across all measures). However, these increases in mental health problems returned to pre-pandemic levels at T3, T4 and T5 and only remained slightly elevated for general anxiety, hyperactivity and prosocial behaviour (see Supplementary Table 15–18 for descriptive statistics, including the sample size for extreme groups, MANOVA results and pairwise comparisons across time points).

Polygenic scores at extremes did not show different trajectories for the low and high extremes groups (± 1 s.d.); these results are presented in Supplementary Fig. 3–8.

### Environmental extremes

We also examined the effects of environmental extremes on mental health outcomes during the COVID-19 pandemic and lockdown. For example, we compared low versus high (±1 s.d.) family SES obtained when the twins were infants. As expected, we found that on average, individuals with lower SES reported worse mental health than individuals with higher SES, although the differences were small (partial eta squared = 0.012; see MANOVA results in Supplementary Table 19). However, we did not see differential trajectories for these groups across the COVID-19 pandemic – that is, there was no moderation between time and SES in explaining mental health outcomes (see Supplementary Fig. 9 and, for MANOVA results, Supplementary Table 19).

We also investigated other possible environmental moderators, including parenthood, having access to green or garden space, having a family member experience job loss, and having extreme financial struggles such as worrying about paying for food. We found little evidence that these environmental factors had an effect on the response of these young adults to the COVID-19 pandemic (Supplementary Figs 10–13).

In addition, we did not observe differences in mental health symptoms between individuals who had COVID-19 diagnoses or COVID-like symptoms at any point during the pandemic (26%) compared with individuals with no symptoms (see Supplementary Fig. 14). Nor did differences emerge between individuals with versus without possible ‘long-COVID’ symptoms, although the possible long-COVID sample was small (*N* = 47; Supplementary Fig. 15).

Finally, we show that the sum score of major family events (infected with COVID-19, admitted to hospital, losing job or loss of family member) was only correlated weakly (*r* = 0.07 on average) with mental health symptoms. A composite score of worries correlated modestly with mental health symptoms (*r* = 0.21 on average). See Supplementary Fig. 16.

## Discussion

Despite the expectation of catastrophic effects of the COVID-19 pandemic on mental health, especially for young people, we found few longer-term consequences, phenotypically or aetiologically. The largest average negative effects – increased hyperactivity and decreased prosocial behaviour – were modest and emerged temporarily during the first month after lockdown (Fig. 2). For prosocial behaviour, the change disappeared entirely 4 months after lockdown; for hyperactivity, the change diminished 7 and 11 months after lockdown. In addition to these modest negative effects, several average changes were in the positive direction, such as decreased conduct problems and decreased emotional problems.

Our LPA (Fig. 3) supported these phenotypic conclusions at the level of individual differences. We did not identify any meaningful subgroups, with one possible exception: one small latent class of 35 individuals showed a sharp rise in self-harm, which continued throughout the pandemic, despite the overall decline in self-harm before and after lockdown (Fig. 2). These findings warrant caution; this self-reported measure of self-harm was reported on a four-point scale and produced a highly skewed distribution. It is also possible that this set of 35 individuals self-selected to the study specifically because of their unusual self-harm results. These results warrant further investigation.

Part of the reason for the perception of the negative effect of COVID-19 on mental health is that reports often focus on statistical significance of mean differences rather than effect size. With a large sample size, such as that of the current study, almost any mean difference will be statistically significant, despite small effect sizes. These findings add to the growing evidence of smaller negative effects on mental health than previously expected.^15–18^ Our results are also in line with reports suggesting that population-level symptoms of mental health increased immediately after lockdown but then decreased again as the pandemic continued.^14,16,65^

An exception is that young adults in England and Wales who reported more mental health problems before the pandemic showed even more problems during the pandemic (Fig. 6). This finding is well aligned with reports that the pandemic was especially detrimental for individuals with existing psychological and psychiatric vulnerabilities.^6,8,22,32^ However, we found that most of these effects were confined to 1 month after lockdown, and mental health generally returned to pre-pandemic levels as the COVID-19 crisis wore on.

Focusing on individual differences rather than average differences enabled several analyses. First, a simple comparison of phenotypic variance across time was illuminating. If individuals responded differently to the COVID-19 crisis, we would expect to see increased variance during the pandemic; however, no increase was observed. Second, even in the absence of phenotypic changes in variance, it is possible that the underlying genetic and environmental causes of variance could have shifted because of the COVID-19 crisis, that is, genetic effects explaining individual differences could have been amplified or reduced; it is also possible that new (innovative) genetic effects could explain some variations in mental health symptoms. However, no systematic changes in heritability were observed (Fig. 5(a)). The most striking genetic result was the average genetic correlation of 0.95 for mental health measures before and during the pandemic (Fig. 5(b)), indicating that on average the genetic factors explaining individual differences in mental health did not change during the pandemic.

Similarly, environmental factors did not moderate the effects of the pandemic. The trajectories of mental health across the year of the COVID-19 crisis did not differ as a function of SES, financial struggles or negative family events. Nor did we find any effect of reported COVID-19 symptoms on mental health.

These findings did not confirm our pre-registered hypotheses (https://osf.io/gzrk3) of changes in mental health symptoms; we did not find evidence for such changes in our sample on average. However, the group of young adults with pre-existing mental health vulnerabilities experienced increased mental health symptoms at the start of the pandemic, with their increased mental health problems subsiding as the pandemic persisted. We conclude that our results speak to the resilience of young people, whose lives were highly disrupted socially and economically by the crisis.

It should be noted that our results are limited to young adults in their twenties and will not necessarily generalise to other ages. Because this age group will be vital to the economy in the coming decades, it is good news for the economy and society if young people come through the pandemic relatively unscathed.

Although the sample was reasonably representative of its cohort, it consisted of twins; furthermore, participants were more likely to be White and were more educated than the UK general population. We also only used self-reported data, so the usual limitations apply here. Attrition and selection bias might have influenced the results. It is well documented that participants in most studies tend to be slightly healthier and more educated than the general population; therefore, the associations observed between study variables might be biased by collider effects.^66^ This bias may have been amplified by data collections happening during the pandemic.^67^ Our sample also experienced attrition, and healthier, more educated participants took part in all data collection waves (Supplementary Table 20). However, the results here remained highly similar whether we used the full data (descriptive statistics) or imputation (LPA) or FIML (LGC) statistical approaches, and our sample obtained at T1, before the pandemic, was reasonably representative of the 1994–1996 birth cohort. Furthermore, our results remain highly similar if we compare descriptive statistics for the whole sample at T1, and when reduced to participants who also participated in T2 data collection (Supplementary Table 21). More research is needed to assess the effects of the COVID-19 crisis on mental health in other populations and age groups.

We conclude that the mental health of young adults in England and Wales has been remarkably resilient to the global pandemic and associated lockdown. Although mental health symptoms worsened immediately after the first lockdown, these effects subsided with time. The effects were stronger for young adults with pre-existing psychological and behavioural problems; however, the worsening symptoms of these individuals also subsided as the pandemic evolved. These conclusions do not mean that the distress and worry felt by many of our participants during this crisis should be dismissed, nor do they mean that there may not be lasting effects of the crisis for the most vulnerable individuals. However, these results could inform preventive interventions, as they indicate that the greatest vulnerabilities were felt at the start of the pandemic. The COVID-19 pandemic is ongoing, and the long-term impact of the pandemic on the mental health of young adults is not known. Studying the psychological vulnerabilities of young adults should remain a research priority, as young adulthood is a tipping point for lifelong psychological problems.

## Data Availability

Data for this study were from TEDS. Researchers can apply for access to the data: http://www.teds.ac.uk/researchers/teds-data-access-policy.

## Supplementary material

For supplementary material accompanying this paper visit https://doi.org/10.1192/bjo.2022.506. click here to view supplementary material
